# Successful treatment of a primary thoracic dumb-bell yolk sac tumor presenting with severe spinal cord compression: Case report

**DOI:** 10.1097/MD.0000000000017610

**Published:** 2019-10-25

**Authors:** Zhenfeng Li, Zhaorui Lv, Qiang Yang, Jianmin Li

**Affiliations:** Department of Orthopedics, Qilu Hospital of Shandong University, Jinan, Shandong Province, China.

**Keywords:** chemotherapy, dumb-bell yolk sac tumor, epidural and extraspinal, needle biopsy, radical resection, spinal cord compression

## Abstract

**Rationale:**

Primary thoracic dumb-bell yolk sac tumor (YST) with both epidural and extraspinal extension is a rare disease with no standard curative managements yet. The objective of this study is to report a primary thoracic dumb-bell YST presenting with severe spinal cord compression successfully treated with posterior-only approach operation, followed by chemotherapy. The management of these unique cases has not been fully documented.

**Patient concerns:**

A 26-mounth-old, previously healthy girl presented with progressive numbness and weakness of the lower extremities. Neurological examination revealed paralysis of both lower extremities, sensory disturbance below T-8 and bladder-bowel dysfunction.

**Diagnosis:**

CT and MRI of spine showed a dumb-bell mass lesion with both epidural and extraspinal extension through enlarged intervertebral foramina and marked spinal cord compression at T7–T9. The AFP level was 13738 ng/ml. Preoperative puncture and Postoperative pathology confirmed the diagnosis of YST.

**Interventions:**

By needle biopsy, we identified the pathological diagnosis is YST. Subsequently, the patient was treated with one-stage posterior-only approach operation, followed by 9 courses of chemotherapy based on cisplatin, bleomycin, etoposide.

**Outcomes:**

The patient has a complete neurologic recovery and remains recurrence free as of more than 2 years after the completion of operation. There were no other complications associated with the operation during the follow-up period.

**Lessons:**

YST should be considered in the range of children with thoracic dumb-bell tumor presenting with spinal cord compression. Needle biopsy is valuable for preoperative diagnosis and design of the treatment strategy. If there is no evidence of CSF spread, metastasis or multiple diseases, it is effective to remove tumors as thoroughly as possible immediately, avoid further nerve injury and conduct enough chemotherapy. This case suggests that this treatment strategy is an effective option for primary YST with both epidural and extraspinal extension and severe spinal cord compression.

## 1 Introduction

Yolk sac tumor (YST), also called endodermal sinus tumor, is a rare malignant germ cell neoplasm that usually occurs in the testis or ovaries of children or young adults. Occasionally, it can affect extragonadal sites involving posterior midline structures in the mediastinum, retroperitoneum, sacrococcygeal region, and intracranially in the suprasellar and pineal regions.^[1]^ Primary YST presenting with both epidural and extraspinal extension is extremely rare. To date, just a few cases of primary YST with both epidural and extraspinal extension have been reported in the literature. We report a case of a primary thoracic dumbbell YST presenting with both epidural and extraspinal extension and severe spinal cord compression from T7-T9. We believe this to be the first reported case of a dumbbell epidural and extraspinal YST presenting with severe spinal cord compression, successfully treated with one-stage posterior-only approach operation and chemotherapy based on cisplatin, bleomycin and etoposide without radiation, with a complete neurologic recovery.

## 2 Case report

### 2.1 History and examination

**Figure 1:**
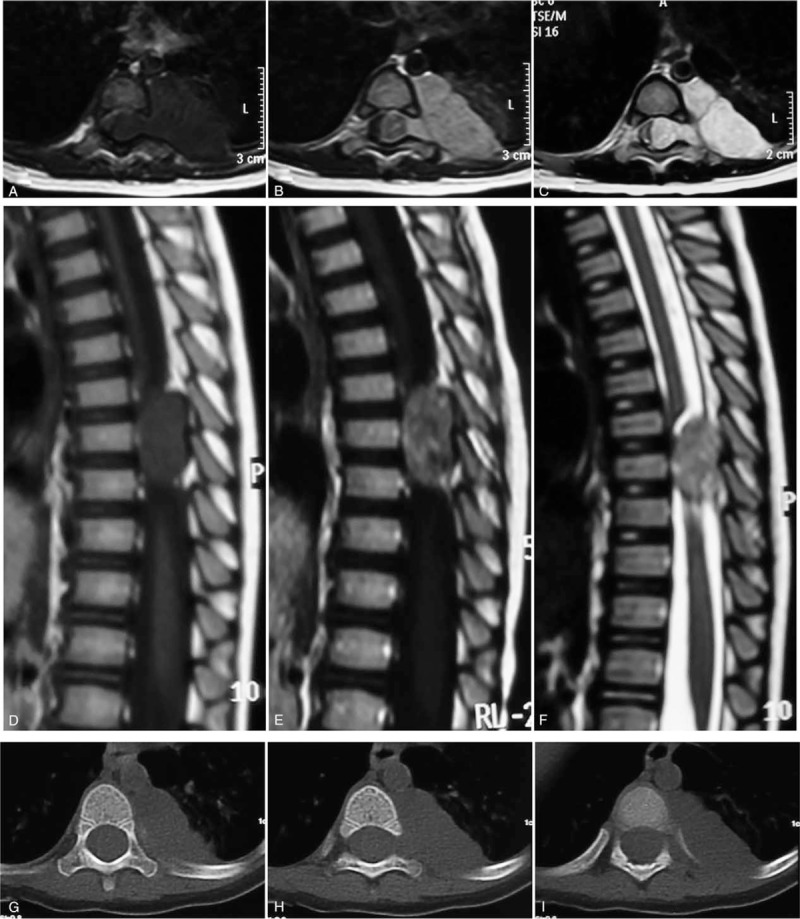
T1-weighted (A, D), Gadolinium-enhanced T1-weighted (B, E), T2-weighted (C, F) MR images, and CT (G, H, I) showing a large extradural and extraspinal mass and marked spinal cord compression at T7–9.

The patient provided written informed consent authorizing use of his protected health information for publication of this report and any accompanying images. A 26-mounth-old, previously healthy girl who had been noticed to fall suddenly when she walked 10 days earlier presented with progressive numbness and weakness of the lower extremities. She could not stand and moved her lower limbs. Neurological examination revealed paralysis of both lower extremities, sensory disturbance below T-8 and bladder-bowel dysfunction. The muscles of lower extremities were graded as 0/5 and the Frankel grade was B. Both knee-jerk and Achilles tendon reflexes were absent. Babinski sign was positive bilaterally. Cranial nerve and upper extremity neurologic functions were normal. Blood tests showed her AFP level were 13,738 ng/ml. β-human chorionic gonadotropin level was normal. CT and MRI were performed immediately and demonstrated a dumb-bell mass lesion with both epidural and extraspinal extension through enlarged intervertebral foramina and marked spinal cord compression at T7–T9. The epidural component occupied three-quarters of the spinal subarachnoid space and part of 8th left rib was invaded by the larger extraspinal component (Fig. 1). The tumor was seen as a zone of iso signal intensity on T1-weighted images (Fig. 1A, D) and a high signal intensity on T2-weighted images (Fig. 1C, F). Enhanced scan suggested evident enhancement (Fig. 1B, E). There is no evidence of CSF spread, metastasis or multiple diseases.

### 2.2 Operation and pathological findings

**Figure 2:**
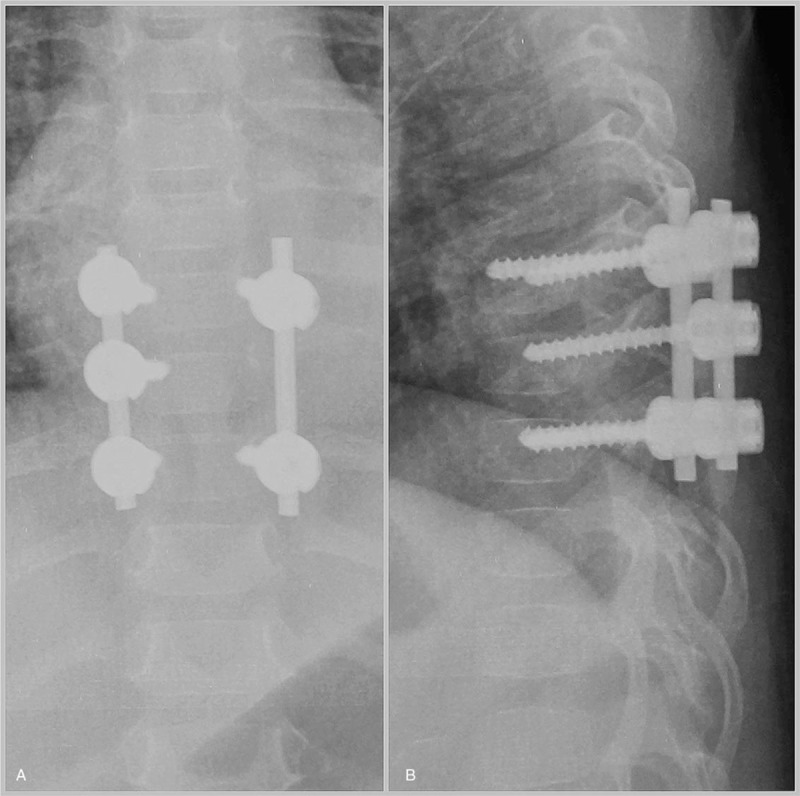
(A) Posteroanterior X-ray image of the thoracic spine obtained postoperatively. (B) Lateral X-ray image of the thoracic spine obtained postoperatively.

**Figure 3:**
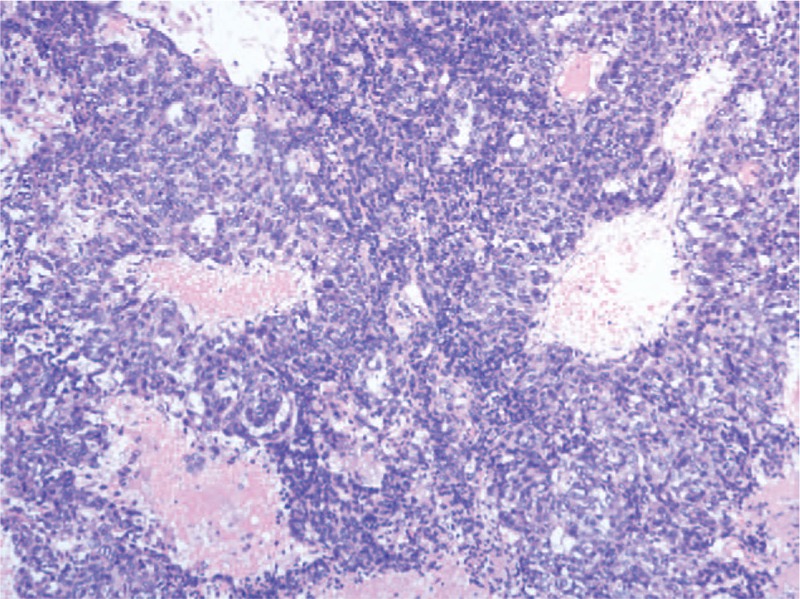
Surgical specimens of yolk sac tumor. H & E staining reveals medium-sized cuboidal or flattened malignant cells in a reticular-microcystic pattern. Schiller-Duval bodies and eosinophilic bodies were present. (×200).

A needle biopsy of the superficial part of the extraspinal mass was performed at prone position by a posterior approach under general anesthesia. Histopathological examination indicated a YST. It was highly reactive for AFP and CK. As the lesion was suspected of being responsible for the progressive paraparesis, emergent posterior decompressive laminectomy from T7-T9 were performed and revealed the epidural tumor. It compressed the spinal cord obviously. The epidural component was removed first. Through the removal of the left T8 vertebral pedicle, zygapophyseal joint and part of 8th left rib, the extraspinal component was exposed and resected thoroughly. Vertebral pedicle screws and bar systems were performed for reconstruction of spinal cord stability (Fig. 2). The final histopathologic findings indicated a YST. (Fig. 3) Immunohistological staining reveals APF, CK, PLAP, EMA were positive, CD117 was probable positive and OCT-4, CD30, S-100 were negative. The proliferative index of Ki-67 was 60% to 70%. Postoperatively, the child recovered lower-extremity function gradually and AFP decreased to 4701ng/ml 2 weeks after the operation.

### 2.3 Postoperative course

Nine cycles of chemotherapy based on bleomycin, etoposide and cisplatin were administered two weeks after the operation. The doses were 3.5 mg/day of bleomycin on days 1 to 2, 50 mg/day of etoposide on days 1 to 5 and 8 mg/day of cisplatin on days 1 to 5. After the completion of second cycle of chemotherapy, AFP dropped to 386ng/ml. AFP levels rapidly decreased to the normal range after completion of 3 cycles. Two months after operation, she could stand up. Neurologically, the child continued to improve and could walk independently and run six months after operation. Internal fixation was removed 1 year after the operation. After more than 2 years of operation, the patient remained without evidence of recurrent and metastasis. The serum AFP and neural function of lower limbs remained normal.

## 3 Discussion

Here, we have reported a case we encountered of primary thoracic dumb-bell YST, which is extremely rare. The patient was successfully treated with needle biopsy, surgery and chemotherapy. Our experience and review of the literature suggest that this treatment strategy might be effective for primary thoracic dumb-bell YST, although further evaluation of the treatment protocol is warranted.

The diagnosis of YST may be inferred from elevated serum AFP that is a very good tumor marker in patients with YST, irrespective of their location. Serial serum AFP determinations can be used for diagnostic purposes, for monitoring the results of treatment, and for early detection of metastases and recurrences.^[2]^

Preoperative needle biopsy is helpful to identify the nature of the tumor and make a treatment plan early. Final diagnosis is dependent on the demonstration of characteristic features by histopathological examination. The histopathological features include Schiller–Duvall bodies, hyalin globules, and immunohistochemical staining for AFP.^[3,4]^

To date, including our case, 5 cases of primary dumb-bell YST causing spinal cord compression have been reported.^[5–8]^ There are no recognized guidelines for the management of dumb-bell YST due to rarity of the disease. Based on published literature, early diagnosis and multimodal treatment with surgical excision, chemotherapy, and radiotherapy may improve the prognosis and limit the morbidity and mortality.

Todani et al^[5]^ reported a 13-month-old male with an approximately 4-week history of complete paraplegia. CT showed a paravertebral solid mass on the right side of the 11th and 12th thoracic vertebrae extending to the 11th rib, aorta, muscle complex of the back, and the intraspinal canal. He was treated with radiation, followed-up by chemotherapy and surgical excision completed by thoracotomy with decompressive laminectomy from the 10th to 12th vertebrae. A pathological diagnosis could not be obtained because the specimen was highly degenerated due to preoperative radiotherapy and chemotherapy. At the age of 34 months, a back tumor recurred at the site of the laminectomy. An immediate biopsy confirmed the lesion to be an YST. He did not recover neurologically.

Resnick et al^[6]^ reported a 21-month-old boy with an approximately 2-week history of flaccid paraplegia. MRI showed a large left suprarenal mass with epidural extension and marked spinal cord compression at T9–L1 from a primary YST. The child underwent an urgent osteoplastic laminotomy and resection. After surgery, he did not recover lower extremity function. He completed first cycle of chemotherapy with bleomycin, VP-16, and cisplatin on postoperative Day 12 and died suddenly 4 days later. The cause of death is unknown.

Pashankar et al^[7]^ reported a 17-month-old boy with a 3-day history of near-complete paraplegia secondary to spinal cord compression from a primary YST. CT showed a large left suprarenal mass with epidural extension and marked spinal cord compression at T10-L1. He was treated with cisplatin-based chemotherapy without laminectomy or radiation therapy. The child had an excellent tumor response and complete neurologic recovery.

Thomas et al^[8]^ reported a 2.5year-old girl with spastic paraparesis and bladder dysfunction. Thoracic X-rays showed a left posterosuperior mediastinal mass with rib erosion. MRI showed that this mass had developed intraspinally between the intervertebral foramina and caused spinal cord compression at T4-T6. Surgical resection via laminectomy was performed in emergency, but the T5 root had to be excised. Pathologic examination showed histologic features of a primary YST. The patient was given chemotherapy for 6 months and is well 2 years later.

In this report, we describe the fifth case of a primary dumb-bell YST presenting with compression. One-stage posterior-only approach was used in this operation of epidural and extraspinal tumors. The advantages of this way are less damage without thoracotomy and avoid related complications. It required high technical requirements relatively. The excision of partial appendix of vertebra influenced the stability of the spine. Therefore, internal fixation using vertebral pedicle screw and bar system was performed.

In patients with YST, the cumulative high-dose chemotherapy with bleomycin, etoposide and cisplatin regimen resulted in excellent overall survival and did not seem to impair ovarian function.^[9]^ Pashankar et al^[7]^ thinks that chemotherapy alone is an alternative to laminectomy or radiation therapy in the management of epidural cord compression from YST and this approach decreases the incidence of severe spinal deformities or instability after laminectomy. It is worth noting that patients who have recent neurologic dysfunction or show rapidly progressive neurologic deficits should be indications for neurosurgical decompression. Neurosurgical decompression was performed if paraplegia had evolved within 72 hours of presentation or if neurologic deterioration occurred while undergoing observation during a 12-hour period.

Mukasa et al^[10]^ think whether irradiation should be performed before or after several courses of chemotherapy is a matter for discussion. Initial irradiation often causes severe myelosuppression and would thus reduce the strength of subsequent intensive chemotherapy. As a result of the complete removal of tumors, we did not carry out radiotherapy.

## 4 Conclusion

In conclusion, YST should be considered in the range of children with thoracic dumb-bell tumor presenting with spinal cord compression. CT and MRI are the first selected imaging examinations for the diagnosis of these tumors. Needle biopsy is valuable for preoperative diagnosis and design of the treatment strategy. Radical resection of the tumor is the key to success. Timely nerve decompression and spinal internal fixation can improve nerve function significantly and reduce the incidence of spinal instability. If there is no evidence of CSF spread, metastasis or multiple diseases, it is effective to remove tumors as thoroughly as possible immediately, avoid further nerve injury and conduct enough chemotherapy.

## Author contributions

**Conceptualization:** Jianmin Li.

**Investigation:** Zhaorui Lv, Qiang Yang.

**Methodology:** Jianmin Li.

**Resources:** Zhenfeng Li, Qiang Yang.

**Supervision:** Jianmin Li.

**Writing – original draft:** Zhenfeng Li, Zhaorui Lv.

**Writing – review & editing:** Zhaorui Lv, Jianmin Li.
